# Combatting psoriasis with nanomedicine: gamma-amino butyric acid-chitosan nanoparticles as a targeted anti-psoriatic therapy

**DOI:** 10.1007/s10787-026-02248-9

**Published:** 2026-04-29

**Authors:** Dina Ibrahim, Alyaa Farid, Mohamed Kishta, Neveen Madbouly

**Affiliations:** 1 Biotechnology Department, Faculty of Science, Cairo University, Giza, 12613 Egypt; 2 Hormones Department, Medical Research and Clinical Studies Institute, National Research Centre, Dokki, Cairo, Egypt; 3 Zoology Department, Faculty of Science, Cairo University, Giza, Egypt

**Keywords:** Psoriasis, Gamma-aminobutyric acid, Chitosan nanoparticles, Drug delivery, Nanomedicine, Imiquimod model

## Abstract

The chronic inflammation and oxidative stress are the most deleterious pathogenic factors of psoriasis (PS). While gamma-aminobutyric acid (GABA) possesses well-documented immunomodulatory and antioxidant properties, its therapeutic potential is limited by bioavailability and targeting. Chitosan nanoparticles (CSNP) offer a promising drug delivery platform to overcome GABA limitations. This study aimed to augment the dermatological efficacy of GABA by encapsulation within CSNPs to overcome its skin penetration limitations, thereby creating an advanced transdermal delivery system for sustained anti-psoriatic local action. TEM revealed spherical, monodisperse GABA-CSNPs. Dynamic light scattering (DLS) confirmed a nanoscale size (57.63 nm), highly positive surface charge (+ 35.93 mV) and excellent colloidal stability. In vitro, GABA-CSNPs demonstrated superior antioxidant (using 2,2-diphenyl-1-picrylhydrazyl (DPPH) assay) and anti-inflammatory (membrane stabilization) activities compared to free GABA or blank CSNPs, beside enhanced biocompatibility (using 3-(4,5-dimethylthiazol-2-yl)-2,5-diphenyltetrazolium bromide (MTT) assay). A PS-like model was induced in rats using 5% imiquimod (IMQ). Animals were divided into five groups: negative control, psoriatic control (PC) (62.5 mg of Aldara® cream) and IMQ groups treated with free GABA ( 200 mg/kg), unloaded CSNPs (100 mg/kg), or GABA-CSNPs (100 mg/kg). The GABA-CSNPs treatment group showed the most significant clinical improvement, reducing scaling and erythema. Mechanistically, therapy restored epidermal architecture, ameliorated oxidative stress by lowering malondialdehyde (MDA) and elevating superoxide dismutase (SOD) and catalase (CAT) levels, and potently suppressed key pro-inflammatory cytokines (IL-1*β*, IL-6 and TNF-*α*) in skin tissue. In conclusion, GABA-CSNPs constitute a novel and highly effective antipsoriatic nanotherapeutic platform. The formulation synergizes the inherent bioactivity of GABA with the enhanced delivery and targeting capabilities of CSNPs, resulting in a potent dual-action therapy that alleviates oxidative damage and modulates the dysregulated immune response central to psoriatic pathology.

## Introduction

Psoriasis (PS) is an autoimmune skin disorder characterized with chronic inflammatory skin condition that affects over 120 million individuals globally and is complicated and (Michalek et al. 2016) lifelong. It has been demonstrated that PS not only causes people to experience pain and itching, but also significantly impairs their psychological health (Strouphauer et al. 2023). Pathological processes are characterized by excessive angiogenesis and vasodilatation, increased proliferation and differentiation of keratinocyte in association with immunological inflammation (Matiushenko et al. 2020). Genetic predisposition, infections, vitamin D deficiency, sun exposure, physical trauma, extreme stress and some drugs are some of the risk factors that may contribute to its development (Rendon and Schäkel 2019). PS has been divided into several categories by the American Academy of Dermatology, including plaquePS (PS vulgaris), eruptive PS, pustular, and erythrodermic (exfoliative PS), inverse PS, or intertriginous psoriasis and eruptive psoriasis. The plaque PS is the most common type that affects about 80% of psoriatic populations (Aijaz et al. 2022). On the clinical level, the psoriatic plaques can be readily recognized by the appearance of the surrounding skin, which include red, swollen regions in the form of oval or circular profusions of white or silvery scales. Psoriatic plaques also have widespread distribution throughout the body, including the oral cavity and genital organs, with severe itching, prolonged duration, difficulty treating, and susceptibility for relapse (Cheng et al. 2024). Symptoms of PS can be exacerbated by lifestyle dynamics like stress, metabolic syndrome, smoking and alcoholic consumption (Rendon and Schäkel 2019). In 10–30% of cases, the long-term consequences of PS can lead to psoriatic arthritis. Psoriatic arthritis is an inflammatory condition that causes cartilage erosion. Prolonged inflammation finally leads to terminal joint damage (Ahmad et al. 2023).

Despite significant progress in PS research over many decades, etiology and pathophysiology underlying this disease remain incompletely elucidated. This knowledge gap represents a pressing need for more effective therapeutic solutions. Current clinical management of PS employs a multi-faceted approach that includes localized treatments, systemic medications, phototherapy, traditional Chinese herbal medicine, combination strategies, and increasingly, targeted biological and small-molecule therapies (Wu et al. 2024).

Gamma-aminobutyric acid (GABA) is a non-proteinogenic amino acid found in diverse organisms such as, animals, plants, yeast and bacteria. In animals, GABA serves as a key signalling molecule during both embryonic and adult neurogenesis, functioning as the primary neurotransmitter in the developing brain (Icer et al. 2024). As the immune and nervous systems engage in bidirectional crosstalk. Locally, the central nervous system (CNS) produces and responds to immune mediators, while the immune system similarly synthesizes and reacts to neuroendocrine signals (Dantzer 2018). GABA as a neurotransmitter exhibits diverse biological activities, including anti-hypertensive, antidiabetic, anticancer, antioxidant, anti-inflammatory, antimicrobial, and anti-allergic properties (Lee and Paik 2017). As well, GABA modulates immune responses by downregulating IFN-*γ* and IL-17 secretion while impairing antigen-presenting cell (APC) function through reduced MHC II and CD80 expression (Tian and Kaufman 2023). On the other hand, GABA enhances immunoregulatory mechanisms by upregulating IL-10 and TGF-*β* (Bajic´ et al. 2020). GABA often plays an epigenetic role in regulating cellular processes such as proliferation, neuroblast migration, and dendritic maturation during wound healing (Han et al. 2007). Previous research indicated that the GABA-A receptor is present in epidermal keratinocytes, where it triggers chloride ion flux and suppresses depolarization (Stoebner et al. 1999). Moreover, other study has shown that GABA may help in maintain skin barrier homeostasis by modulating the GABA-A receptor (Denda et al. 2002). In human skin models, GABA improved the skin’s barrier function (Cagno et al. 2010), skin elasticity (Uehara et al. 2017; Ito et al. 2007) via the expression of crucial genes such as filaggrin, human beta-defensin-2 (HBD-2), human hyaluronan synthase (HAS1) involved in the manufacture of filaggrin, hyaluronic acid protein and elastin synthesis. GABA also inhibits matrix metalloproteinase − 1 (MMP − 1) and regulation of type I collagen it improves the function of the skin’s barrier. PS, a chronic inflammatory skin disorder, has been linked to dysregulation in the GABAergic system. Experimental evidence indicates elevated expression of GABA ligands and GABA-A receptors in psoriatic lesions, implicating their role in disease pathogenesis and itch mediation (Chen et al. 2021; Nigam et al. 2010). A clinical study reveal that GABA deficiency is prevalent among PS patients, with 85% of plaque PS cases and 90% of arthropathic PS cases exhibiting significantly reduced GABA levels in serum (Matiushenko et al. 2020). This deficiency suggests a potential pathophysiological link between impaired GABAergic signaling and psoriatic disease manifestations. Given these findings, GABA-based therapeutics may hold promise as a novel strategy for managing and potentially ameliorate PS. However, the pharmacokinetics of oral GABA probably hinder its bioactive advantages due to low bioavailability and absorption as well as a quick clearance rate within about 20 min (Oketch-Rabah et al. 2021). The transdermal delivery technique enables GABA to skip the metabolic gauntlet of the digestive tract and enter the bloodstream together with the sustained release of GABA over a prolonged period, usually 24 h (Ruggiero 2024).

Chitosan (CS), a cationic biopolymer derived from the partial deacetylation of chitin, is sourced primarily from fungal cell walls and crustacean exoskeletons (e.g., shrimp and crabs) (Matouri et al. 2024). As an FDA-approved biomaterial, CS has biodegradable, and biocompatible, anti-inflammatory properties, serum stability, solubility, non-immunogenicity, appropriate pharmacokinetics and pharmacodynamics, properties, making it valuable for tissue regeneration and drug delivery applications as an appropriate alternative to overcome many drug limitations (Shariatinia 2019; El-Saadony et al. 2025). Numerous studies have employed CS to generate safe delivery systems for pharmaceuticals, plant extracts, microbes, and their soluble constituents (Gonciarz et al. 2025). CSNPs is a multipurposepolymer has broad biomedical utility, including: wound dressing (Che et al. 2024), tissue engineering (Cui et al. 2019) regenerative medicine (Meyer-Déru et al. 2022), food additive (Niculescu and Grumezescu 2022), biosensors (Zhong et al. 2011), antimicrobial activity (Guan and Feng 2022), imaging (Li et al. 2022), treatment of diseases (Güngör et al. 2021). CSNPs based therapies have proven wound healing outcomes (Che et al. 2024; Feng et al. 2021; Ali Khan et al. 2020; Liu et al. 2016; Patrulea et al. 2015). CSNPs were also efficient in anti-psoriatic treatments (Yeo and Kim 2025). Furthermore, CS possesses unique immunological characteristics, such as the ability to regulate pro-inflammatory cytokines and promote tissue granulation by recruiting fibroblasts so it had great potential in skin-related conditions and specially PS (Cheng et al. 2024; Khalil et al. 2025; Sheikh et al. 2023).

The principal objective of this study was to augment the dermatological efficacy of GABA by overcoming its skin permeation limitations, thereby improve its potential as a novel anti-psoriatic agent via enhanced antioxidant and anti-inflammatory activities. GABA was encapsulated within CSNPs to function as an advanced transdermal delivery system. The formulated GABA-CSNPs were systematically characterized for their physicochemical properties, subjected to stability profiling, and evaluated for in vitro release and bioactivity. Furthermore, the therapeutic efficacy of the optimized nano-formulation was systematically investigated through an in vivo study, utilizing a PS-like model in Sprague–Dawley rats to assess its capacity to ameliorate psoriatic pathology.

## Materials and methods

### Preparation of GABA-CSNPs

CS (medium molecular weight, ≥ 75% deacetylation, medium molecular weight), GABA and sodium tripolyphosphate (TPP) were purchased from Sigma-Aldrich, USA. All chemicals were of analytical grade and used without further purification. GABA-CSNPs were prepared using the ionic gelation method, based on the electrostatic interaction between positively charged CS and negatively charged TPP (Shilpa et al. 2012). Briefly, CS was dissolved in 50 mL 1% (v/v) acetic acid solution under magnetic stirring to obtain a clear solution with concentration of 1 mg/mL. GABA (20 mg) was then dissolved in deionized water and added to the CS solution under continuous stirring. Subsequently, an aqueous solution of TPP (0.1% w/v) was added dropwise into the CS-GABA mixture under constant stirring (500–1000 rpm) at room temperature. The formation of nanoparticles occurred spontaneously due to ionic cross-linking between the NH_3_^+^ groups of chitosan and the P_3_O_10_^5^⁻ groups of TPP. The resulting nanoparticle suspension was stirred for an additional 30–60 min to ensure complete particle formation and GABA encapsulation. Finally, the suspension was centrifuged (e.g., 10,000–15,000 rpm, 20–30 min) to separate the nanoparticles, which were then washed with deionized water to remove un-encapsulated GABA and freeze-dried for further characterization.

### Physicochemical characterization of GABA-CSNPs

Morphological examination of GABA-CSNPs was performed by transmission electron microscopy (TEM) (JEM-1400 Flash, JEOL, Watchmead, UK). Particle size distribution and polydispersity index were determined by dynamic light scattering (DLS), where nanoparticle suspensions were analyzed at a fixed scattering angle after appropriate dilution in deionized water (Zetasizer Nano ZS90; Malvern Instruments Limited, Malvern, UK). Surface charge measurements were conducted using electrophoretic light scattering to determine zeta potential, with samples properly equilibrated in a clear disposable zeta cell. For drug loading capacity (DL%) and encapsulation efficiency (EE%) calculations, the free GABA content in supernatants obtained after nanoparticle centrifugation is quantified using ultraviolet–visible (UV–Vis) spectrophotometry at the characteristic wavelength of GABA, with appropriate standard curve preparation according to the following equations (Abd El-Ghaffar et al. 2017):

$${\mathrm{GABA}}\;{\mathrm{loading}}\;{\text{capacity }}\left( {{\mathrm{DL}}\% } \right) = \frac{{{\text{Weight of the GABA in nanoparticles}}}}{{{\text{Weight of the yield nanoparticle}}}}$$

$${\text{GABA encapsulation efficiency}} \left( {{\text{EE\% }}} \right) = \frac{{\text{Weight of the GABA in nanoparticles}}}{{\text{weight of the intial GABA}}}$$

In vitro GABA release study was performed by dialyzing nanoparticle suspensions against phosphate buffer saline (pH 7.4) at physiological temperature, with aliquots withdrawn at predetermined time intervals (0.5, 1, 2, 4, 8 and 16 h) for GABA quantification (Gómez-Lázaro et al. 2024). Stability studies involve monitoring particle size, zeta potential, and drug content over time. All characterization experiments were performed in triplicate to ensure reproducibility of the results.

### In vitro characterization of GABA-CSNPs

The antioxidant activity of the samples was evaluated using the DPPH (2,2-diphenyl-1-picrylhydrazyl) free radical scavenging assay according to Kedare and Singh (Kedare and Singh 2011). Briefly, serial concentrations of GABA, CSNPs, and GABA-CSNPs (1000–1.95 µg/mL) were mixed with a methanolic DPPH solution 1 mL (0.1 mM) and incubated in the dark for 30 min at room temperature. The absorbance was then measured at 517 nm using a microplate reader, with ascorbic acid serving as the positive control. The percentage of DPPH radical scavenging activity was calculated relative to a blank control according to the following formula,

$$\begin{gathered} {\text{DPPH scavenging activity }}\left( {{\% }} \right){ = } \hfill \\ \left( {{\text{Absorbance of control - }} {\text{absorbance of sample}}} \right)/{\text{Absorbance of control}} \times {100} \hfill \\ \end{gathered}$$

For the cytotoxicity assessment, the MTT (3-(4,5-dimethylthiazol-2-yl)-2,5-diphenyltetrazolium bromide) assay was performed according to Zhao et al. (Zhao et al. 2023) on cultured human keratinocytes (HaCaT) (Cell Lines Service GmbH, Germany). Cells were seeded in 96-well plates (1 × 10^5^ cells per mL) and cultivated in a cell incubator (5% CO_2_ at 37 °C for 24 h). Then the cells were treated with serial dilutions (1000–31.5 µg/mL) of the test samples from free GABA, CSNPs and GABA-CSNPs for 24 h. Following this, 100 μL MTT solution (1 mg/mL in PBS) was added to each well and further incubated for 4 h at 37 °C. The formed formazan crystals were dissolved in DMSO (100 μL/well), and the absorbance was measured at 570 nm using a microplate reader. Cell viability was expressed as a percentage relative to untreated control cells as follows:

$${\text{Cell viability }}\left( \% \right) = \frac{{\text{Optical density of test sample - Optical density of blank}}}{{\text{Optical density of control sample - Optical density of blank}}} \times 100$$

The hemolysis inhibition activity, as indicator of anti-inflammatory ability, was conducted based on membrane stabilization assay (Tawfik and Farid 2025), by incubating freshly prepared rat red blood cells (RBCs) (40% v/v) with different concentrations (1000–100 µg/mL) of the test samples and 2 mL/well of distilled H_2_O or PBS (as hypotonic and isotonic solutions, respectively) at 37 °C for 1 h. After centrifugation, the supernatant’s absorbance was measured at 540 nm to determine hemoglobin release. PBS and distilled water served as negative and positive controls, respectively. The percentage hemolysis was calculated by comparing the sample absorbance values with the positive control as indicated in formula below. All experiments were performed in triplicate to ensure reproducibility of the results.

$${{\% Hemolysis Inhibition}} = 1 - \left( {{\mathrm{Abs}}_{{{\mathrm{hypo}}}} - {\mathrm{Abs}}_{{{\mathrm{iso}}}} } \right)/\left( {{\mathrm{Abs}}_{{{\mathrm{control}}}} { } - {\mathrm{Abs}}_{{{\mathrm{iso}}}} } \right) \times 100$$

where Abs_hypo_, Abs_iso_, and Abs_control_ represent the absorbance in the hypotonic, isotonic, and control solutions, respectively.

### Animal study design and experimental protocol

Twenty-five healthy male Sprague–Dawley rats (eight weeks old, 160–170 g body weight) were procured from the National Organization for Drug Control and Research (NODCAR), Giza, Egypt and acclimatized under standardized laboratory conditions: a temperature-controlled environment (22 ± 2 °C), regulated humidity, and a 12 h light/dark cycle. All experimental procedures adhered to the ARRIVE (Animal Research: Reporting of In Vivo Experiments) guidelines and were approved by the Institutional Animal Care and Use Committee (IACUC) of Cairo University (Approval No.: CUIF2824).

### PS induction and treatment protocol

-like skin inflammation was induced following the Horváth et al. (Horváth et al. 2019) protocol. Briefly, after shaving the dorsal skin using a depilatory cream (EVA Cosmetics, Egypt), 62.5 mg of Aldara® cream (5% imiquimod, MEDA Pharmaceuticals, Sweden) was applied topically for 10 consecutive days to establish psoriatic lesions. Following successful induction, therapeutic interventions (as free GABA, unloaded CSNPs, or GABA-CSNPs) were administered topically twice daily for 14 consecutive days. The rats were randomly allocated into five experimental groups (n = 5/group) including: Group I (Healthy Control, HC): Untreated, non-psoriatic rats. Group II (Psoriatic Control, PC): Rats with Aldara®-induced PS, left untreated. Group III (GABA-Aldara® induced PS): Psoriatic rats treated with GABA (200 mg/kg). Group IV (CSNPs-Aldara® induced PS): Psoriatic rats treated with CSNPs (100 mg/kg). Group V (GABA-CSNPs-Aldara® induced PS): Psoriatic rats treated with GABA-CSNPs (100 mg/kg). The GABA dose utilized in this study was determined based on the findings of a 28-day sub-chronic oral toxicity study conducted by Hayami et al. (Hayami et al. 2005) and a study by the Japan Food Research Laboratories (JFRL) (Pharma Foods International Co., Ltd. 2002) in an earlier acute toxicity.

### Sample collection and processing

On the 25th day of the experiment, rats were anesthetized via intraperitoneal injection of pentobarbital sodium (50 mg/kg). Skin tissue samples were excised from the dorsal region and homogenized in ice-cold phosphate-buffered saline (PBS). The homogenates underwent two freeze–thaw cycles to ensure complete cellular lysis, followed by centrifugation at 1200 rpm for 15 min to obtain clear supernatants for further biochemical and histological analyses (Salkovskiy et al. 2025).

### In vivo anti-PS effects and histopathological examination of GABA-CSNPs

Dorsal skin erythema, desquamation (scaling) and induration (thickness) were recorded during the experimental period at day 5, 10, 15, 20 and 25. A scale from 0 to 4; based on the Psoriasis Area Severity Index (PASI) as 0; absent; 1, mild; 2, moderate; 3, severe; 4, very severe; sign (Boehncke and Schön 2015). A comprehensive histopathological examination was conducted by an experienced pathologist under blinded conditions to ensure unbiased evaluation of treatment effects. Skin tissue specimens were carefully fixed in 10% neutral buffered formalin for 24 h to preserve cellular architecture. Following fixation, samples underwent gradual ethanol dehydration (70% to absolute alcohol) and were subsequently cleared in xylene to prepare for paraffin embedding. Tissues were then embedded in high-quality paraffin blocks using standardized protocols to maintain structural integrity. Using a precision microtome, 4 μm-thick sections were obtained and mounted on glass slides. For microscopic evaluation, sections were stained with hematoxylin and eosin (H&E) (Bancroft and Gamble 2013).

### In vivo antioxidant and anti-inflammatory activities of GABA-CSNPs

The in vivo antioxidant efficacy of GABA, CSNPs, and GABA-CSNPs was assessed by quantifying oxidative stress markers and antioxidant enzymes in skin tissue homogenates. Lipid peroxidation, an indicator of oxidative damage, was measured via malondialdehyde (MDA) levels using a rat-specific ELISA kit (MBS268427, MyBioSource, USA). Concurrently, the antioxidant defence system was evaluated by determining the activity of superoxide dismutase (SOD) and catalase (CAT), critical enzymes in neutralizing reactive oxygen species (ROS), using a corresponding ELISA kit (MBS036924 and MBS2540413, MyBioSource, USA). These measurements collectively provided insights into the ability of the formulations to mitigate oxidative stress in psoriatic skin.

To assess the anti-inflammatory activity, the levels of key pro-inflammatory cytokines, interleukin-1 beta (IL-1*β*), IL-6 and tumor necrosis factor-alpha (TNF-*α*), were quantified in skin homogenates using rat-specific ELISA kits (E-EL-R0012, E-EL-R0015 and E-EL-R2856, Elabscience, USA). These cytokines are pivotal mediators of inflammation in PS, and their suppression would indicate the therapeutic potential of the tested formulations. By integrating oxidative stress and inflammatory markers, this comprehensive analysis elucidated the dual antioxidant and anti-inflammatory mechanisms of GABA-CSNPs in alleviating psoriatic symptoms. All assays were performed in accordance with manufacturer protocols, ensuring reproducible and standardized results.

### Statistical analysis

All statistical analyses were conducted using GraphPad Prism 8.0.2 (GraphPad Software, Inc., La Jolla, CA). Data are expressed as mean ± standard deviation. Differences between two experimental groups were evaluated using Student’s t-test, while comparisons among multiple groups were performed with one-way analysis of variance (ANOVA) followed by Post-hoc comparisons using the Tukey HSD test. A p-value less than 0.05 was considered statistically significant. All in vitro and in vivo experiments were performed in triplicate.

## Results

### Physicochemical characterization of GABA-CSNPs

**Fig. 1:**
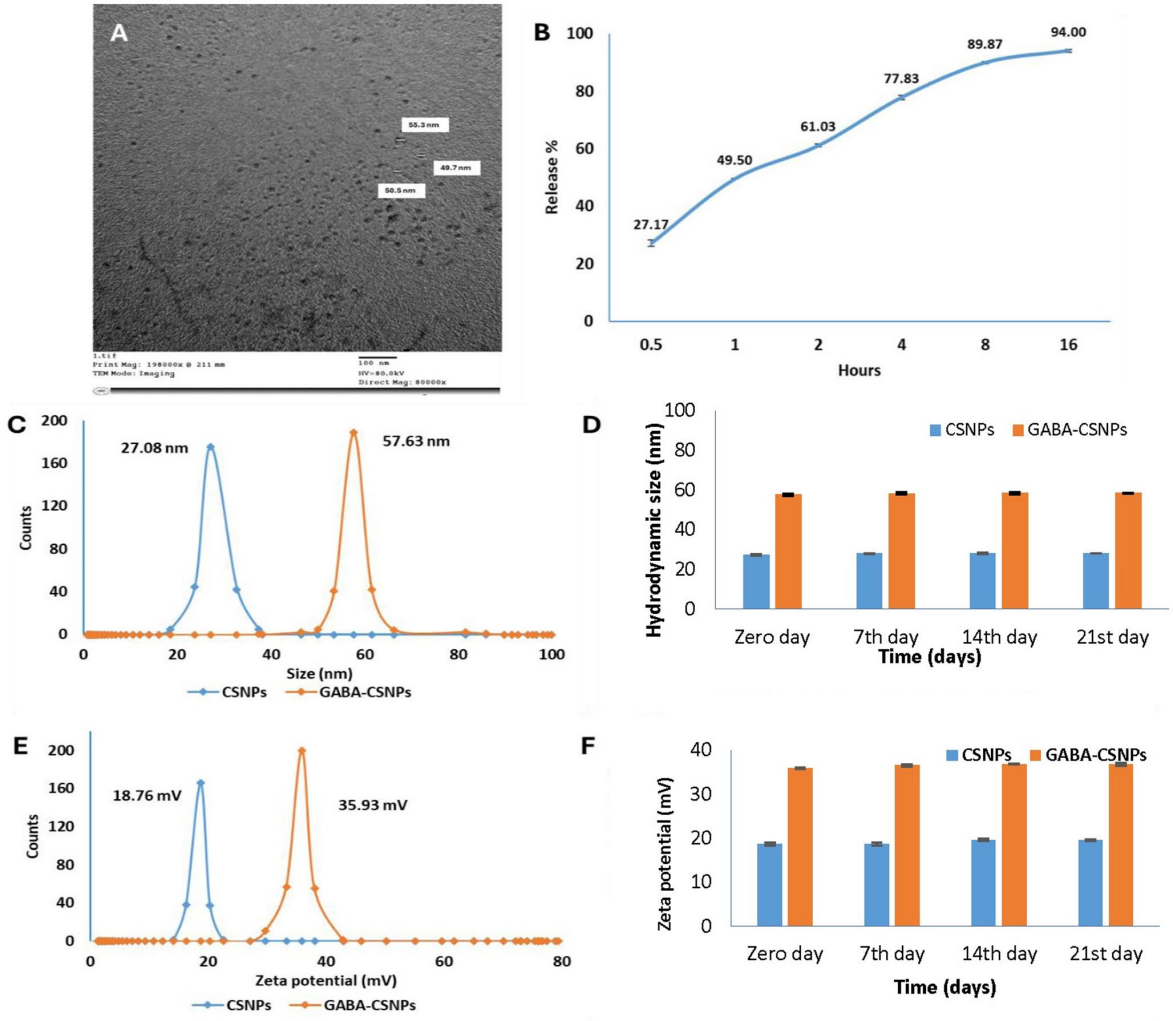
Physical characteristics of GABA-CSNPs showing TEM image **(A)**, in vitro release % **(B)**, hydrodynamic size after preparation **(C) **and along 21-day period **(D)** and surface charge (zeta potential) after preparation **(E)** and along 21-day period **(F)**

The TEM analysis revealed well-defined, spherical morphology of the GABA-CSNPs with a uniform size distribution (Fig. 1A). The measured particle diameters were 49.7 nm, 50.5 nm, and 55.3 nm, demonstrating good consistency in nanoparticle size. The image clearly showed the discrete nature of the nanoparticles without significant aggregation. These results corroborated the successful formation of monodisperse GABA-CSNPs with controlled size parameters suitable for biomedical applications. The synthesized GABA-CSNPs demonstrated DL% of 26.60 ± 0.46 with EE% about 71.83 ± 1.26, indicating a highly efficient and promising system for GABA delivery.

GABA’s in vitro release profile from CSNPs showed a dual release pattern, with a burst release at the beginning and a steady release of the substance after that (Fig. 1B). About 27.17% of GABA was released in the first 0.5 h, demonstrating the quick diffusion of drug molecules attached to the surface. The release rate rose sharply to 49.50% after 1 h and to 61.03% after 2 h, indicating that the drug particles were still dissolving close to the nanoparticle surface. The significant drug release from the polymeric matrix was demonstrated by the release of 77.83% of GABA after 4 h. With a cumulative release of 89.87% at 8 h and a nearly full release of 94.00% at 16 h, the release profile thereafter progressively plateaued. This release pattern demonstrated that GABA-CSNPs have both immediate and delayed release properties, which may make them appropriate for uses where rapid onset and sustained drug administration are necessary.

The particle size distribution of CSNPs and GABA-CSNPs was analysed using DLS (Fig. 1C). The results revealed that CSNPs exhibited a smaller average hydrodynamic diameter of 27.08 nm, while GABA-CSNPs showed a larger size of 57.63 nm, indicating that GABA incorporation increased the nanoparticle size due to drug encapsulation and possible changes in surface morphology.

The stability of the nanoparticles was assessed over a 21-day period by monitoring their size at different time points. As shown in Fig. 1D, CSNPs maintained consistent sizes, measuring 27.3 nm at day zero and showing minimal variation, with values of 28.1 nm, 28.2 nm, and 28.2 nm on the 7th, 14th, and 21st days, respectively. Similarly, GABA-CSNPs demonstrated excellent stability, with an initial size of 57.6 nm and only slight increases to 58.2 nm, 58.3 nm, and 58.3 nm over the same period. These findings confirmed that both CSNPs and GABA-CSNPs possessed good colloidal stability, with no significant aggregation or size alteration during storage (Fig. 1D).

The zeta potential measurements of nanoparticles were conducted to evaluate their surface charge and colloidal stability. As shown in (Fig. 1E), CSNPs exhibited a zeta potential of 18.76 mV, while GABA-CSNPs displayed a higher surface charge of 35.93 mV. The increased zeta potential of GABA-CSNPs was attributed to the additional positive charges introduced by the incorporation of GABA, which enhanced the electrostatic stabilization of the nanoparticles. The stability of the nanoparticles was further assessed by monitoring their zeta potential over a 21-day period (Fig. 1F). The results demonstrated that CSNPs maintained consistent surface charges, with values of 18.6 mV, 18.7 mV, 19.6 mV, and 19.6 mV on days 0, 7, 14, and 21, respectively. Similarly, GABA-CSNPs showed excellent stability, with zeta potentials of 35.9 mV, 36.5 mV, 36.9 mV, and 36.8 mV over the same time frame. These findings indicated that both CSNPs and GABA-CSNPs possessed stable surface charges, which were critical for preventing particle aggregation and ensuring long-term colloidal stability in suspension. The minimal variations in zeta potential over time further confirmed the robustness of the nanoparticle formulations.

### In-vitro characterization of GABA-CSNPs

**Fig. 2:**
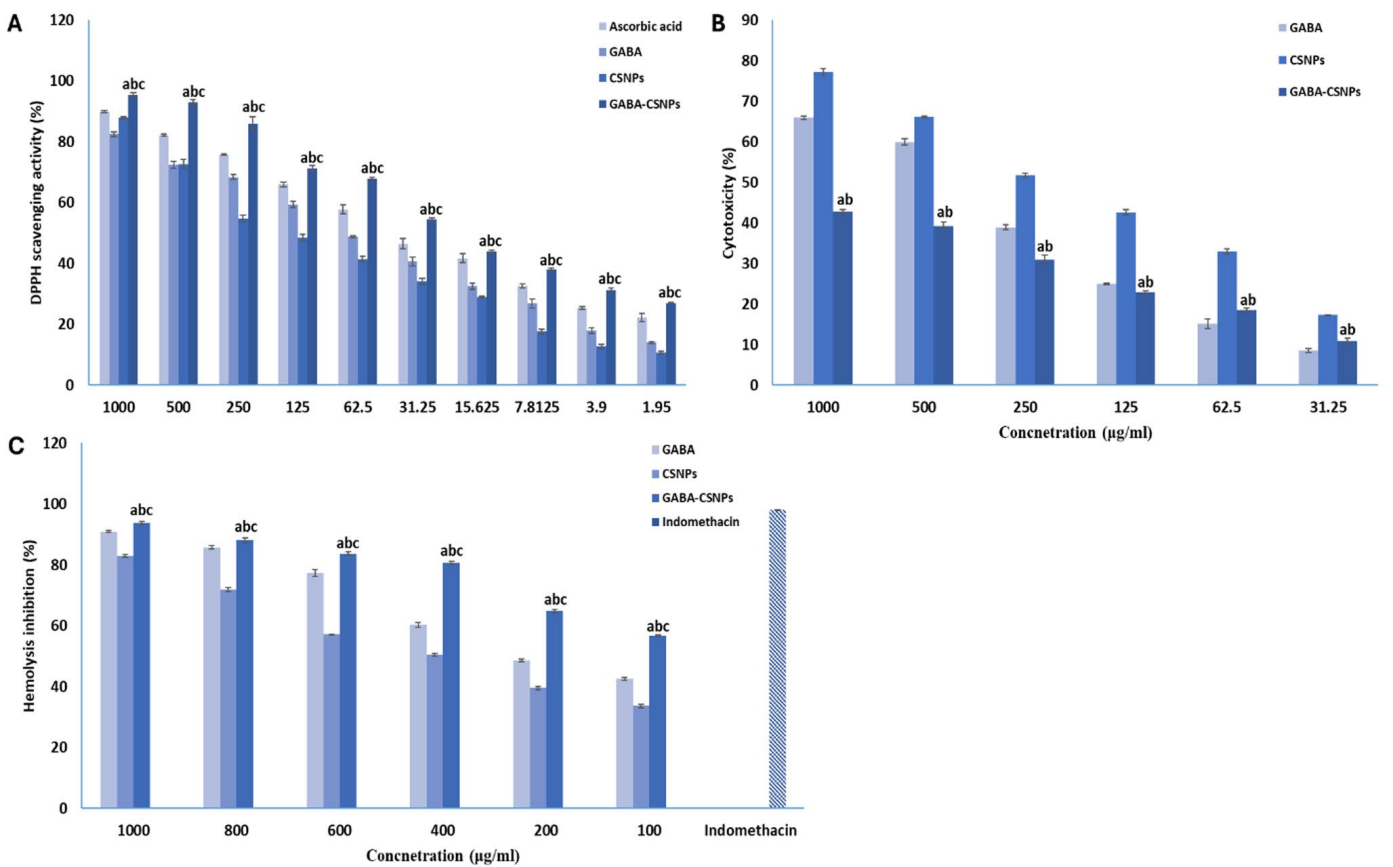
In vitro characterization of GABA, CSNPs and GABA-CSNPs showing the antioxidant activity using 2,2-diphenyl-1-picrylhydrazyl (DPPH) assay **(A)** where a, b, and c indicated significance (*P* < 0.05) with respect to ascorbic acid, GABA, and CSNPs; the cytotoxicity activity using 3-(4,5-dimethylthiazol-2-yl)-2,5-diphenyltetrazolium bromide (MTT) assay **(B)** where a and b indicated significance (*p* < 0.05) with respect to GABA, and CSNPs; the anti-inflammatory activity (hemolysis inhibition assay) **(C)** where a, b, and c indicated significance (*P* < 0.05) with respect to indomethacin, GABA, and CSNPs

The antioxidant activity of GABA-CSNPs was evaluated and compared with pure GABA, unloaded CSNPs, and ascorbic acid (control) using a concentration-dependent DPPH assay. The results demonstrated a dose–response relationship for all tested compounds, with GABA-CSNPs showing significantly enhanced antioxidant capacity compared to both free GABA and CSNPs across all concentrations tested (1.95–1000 μg/mL). At the highest concentration (1000 µg/mL), ascorbic acid exhibited strong (89.9%) antioxidant effect as expected, but GABA-CSNPs displayed superior (95.4%) free radical scavenging activity relative to their individual components free GABA and CSNPs (82.5 and 87.9%; respectively) suggesting a synergistic effect between GABA and the CSNPs (Fig. 2A).

The cytotoxic effects of GABA, CSNPs, and GABA-CSNPs revealed concentration-dependent effects on HaCaT cell viability across a range of concentrations (31.25–1000 μg/mL). Based on the cell viability values, the GABA-CSNP formulation demonstrates a significant reduction in cytotoxicity (42.77%) compared to both its individual components (GABA, 65.90 and CSNPs, 77.07%). GABA-CSNPs recorded IC50 = 1091.49 μg/mL while GABA and CSNPs’s IC50 was 576.21 and 366.73 μg/mL; respectively. These results confirmed the excellent biocompatibility of the GABA-CSNP formulation, supporting its potential for therapeutic applications where minimal cellular toxicity is essential (Fig. 2B).

The anti-inflammatory activity of GABA-CSNPs was evaluated in comparison with free GABA, CSNPs, and indomethacin (reference drug) across a concentration range of 100–1000 μg/mL. The results demonstrated that GABA-CSNPs exhibited significantly enhanced anti-inflammatory effects compared to both free GABA and CSNPs at all tested concentrations. While indomethacin showed the strongest anti-inflammatory activity as a standard NSAID, GABA-CSNPs displayed a concentration-dependent response that was markedly greater to its individual components (Fig. 2C).

### Effect of GABA-CSNPs on symptoms of Aldara®-induced PS model and dorsal skin histopathology

**Fig. 3:**
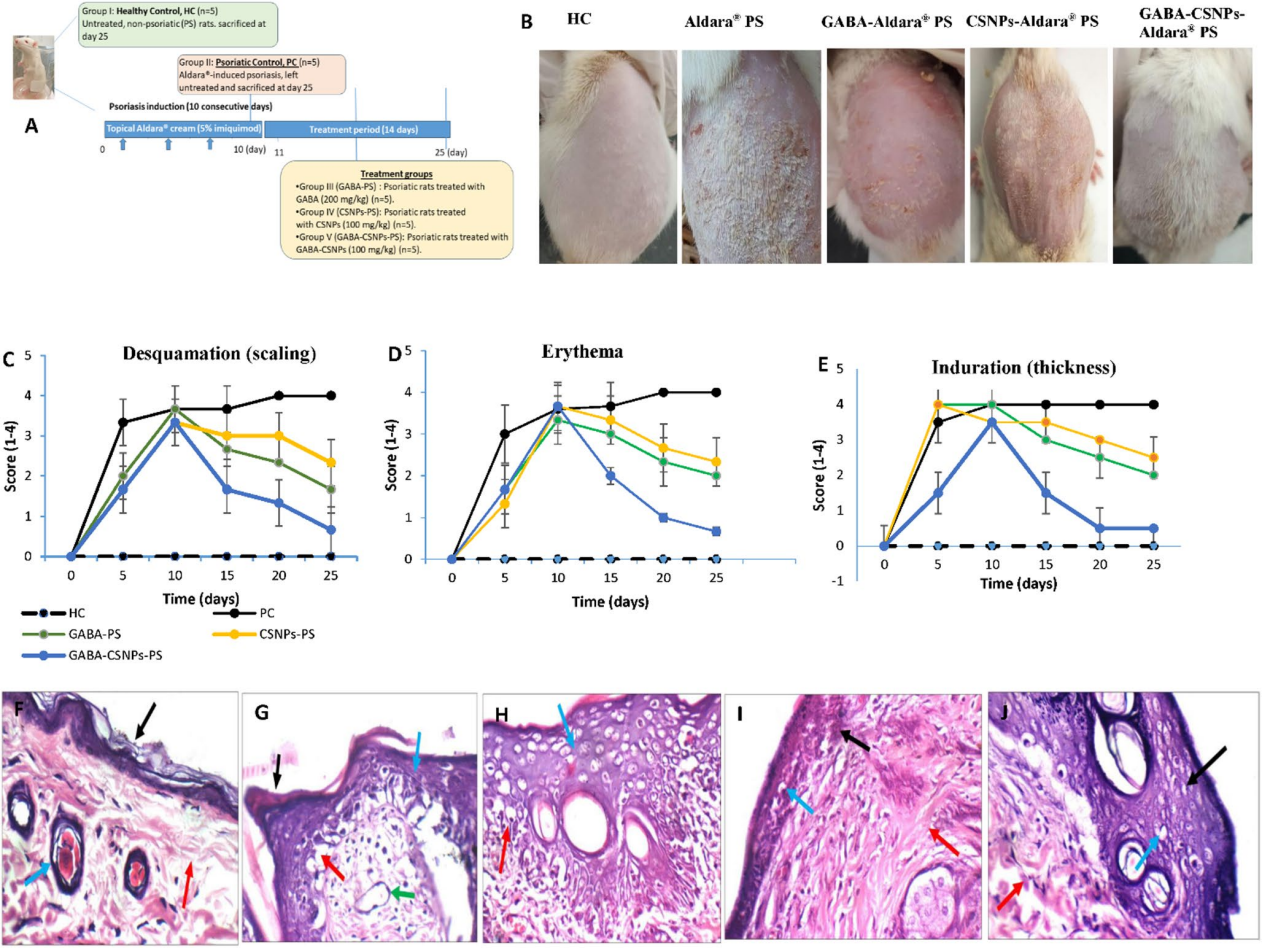
Effect of GABA-CSNPs on symptoms of Aldara®-induced PS model and dorsal skin histopathology. The experimental design **(A)**. Photographs of dorsal skin showing the psoriatic model site of the different experimental groups in Sprague–Dawley rats (day 25) **(B)** to study the PASI score was applied to measure the disease severity by desquamation (scaling) **(C)**, erythema **(D)**, and induration (thickening) **(E)**. Histopathological results in the dorsal skin tissues of rats in different groups using haematoxylin and eosin (H&E) sections showing intact keratinized epidermis (black arrow), dermis with average hair follicles (blue arrow), and average collagen (red arrow) in healthy control (HC) group (X 400) **(F)**, parakeratosis (black arrow), epidermis with thin granular cell layer (blue arrow), marked spongiosis (red arrow), and marked superficial peri-vascular inflammatory infiltrate (green arrow) in psoriatic control (PC) group (X 400) **(G)**, marked diffuse epidermal hyperplasia (blue arrow), and mild spongiosis (red arrow) in GABA-Aldara® induced PS group (X 400) **(H)**, marked focal epidermal hyperplasia (blue arrow), mild spongiosis (blue arrow), and dermis with average collagen (red arrow) in CSNPs-Aldara® induced PS group (X 400) **(I)**, intact keratinized epidermis, mild focal epidermal hyperplasia (black arrow), mild spongiosis (blue arrow), and dermis with average collagen (red arrow) in GABA-CSNPs-Aldara® induced PS (X 400) **(J)**

To explore the antipsoriatic effect of GABA-CSNPs, an animal model of PS was established using Aldara® cream (5% imiquimod) for 10 consecutive days (Fig. 3A). The dorsal skin showed extensive scale formation and marked erythema and noticeable thickened in PC group confirming the PS-like model (Fig. 3B). The score of skin desquamation (scaling) (Fig. 3C), erythema (Fig. 3D) and the induration (thickness) (Fig. 3E) in each group increased with time in the first 10 days. From day 10, all indexes in all intervention groups started to going down, but the extent varied with type of treatment. PS symptoms were reduced after treatment with GABA and CS groups compared to the model group (PC). Notably, the GABA-CSNPs developed the strongest ability to lower these indexes, ameliorated the erythema with reduced wrinkles and skin thickness, the psoriasis symptoms showing a trend similar to that observed in the HC group.

Histological analysis (Fig. 3F–J) by H&E staining of the skin tissues further confirmed these results. PC group, successful PS-like condition was established as increased skin thickness, parakeratosis and hyperkeratosis of the stratum corneum together with marked superficial perivascular inflammatory cell infiltration and spongiosis in dermis. After 14 days of GABA-CSNPs treatment, skin tissue showed decreased thickness with intact keratinized epidermis, mild focal epidermal hyperplasia, mild spongiosis, and dermis with average collagen.

### Effect of GABA-CSNPs on in vivo antioxidant and anti-inflammatory activities

**Fig. 4:**
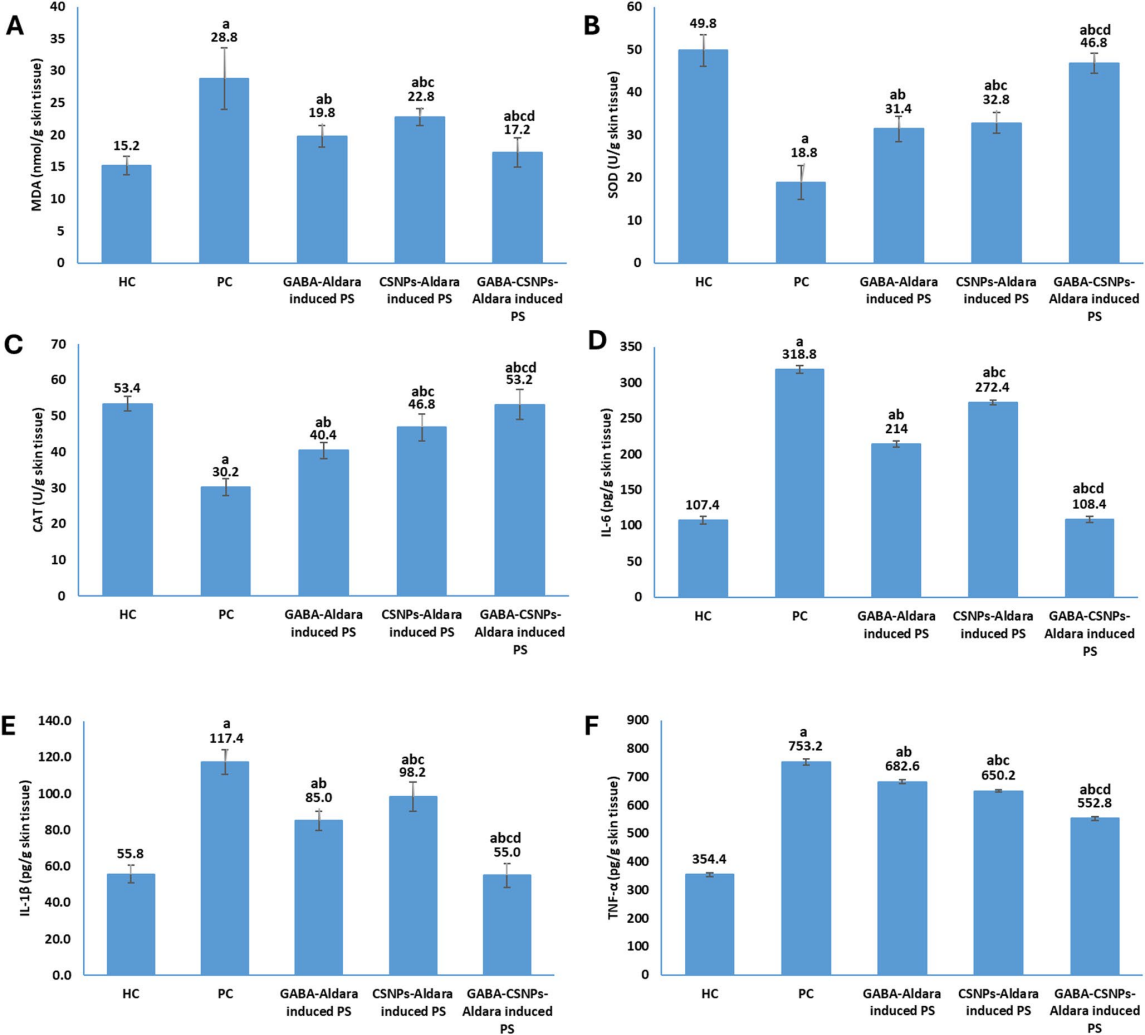
In vivo antioxidant and anti-inflammatory activities of GABA, CSNPs and GABA-CSNPs showing levels of malondialdehyde (MDA) **(A)**, superoxide dismutase (SOD) **(B)**, catalase (CAT) **(C)**, interleukin (IL)-6 **(D)**, IL-1*β* **(E)** and tumor necrosis factor (TNF)-α **(F)**. Results were represented as mean ± SD, where a, b, c and d indicated significance (*p* < 0.05) with respect to healthy control (HC), positive control (PC), GABA treated rats with Aldara®-induced PS(GABA-Aldara® induced PS) and CSNPs treated rats with Aldara®-induced PS (CSNPs-Aldara® induced PS)

The results demonstrated significant differences in oxidative stress and inflammatory markers across the experimental groups. Oxidative stress markers of the PC group exhibited significantly increased lipid peroxidation (MDA: 28.8 nmol/g skin tissue) and decreased antioxidant capacity (SOD: 18.8 U/g skin tissue and CAT: 30.2 U/g skin tissue) relative to HC (MDA: 15.2 nmol/g skin tissue, SOD: 49.8 U/g skin tissue and CAT: 53.4 U/g skin tissue) (*p* < 0.05). GABA-CSNPs treatment effectively restored these parameters to near-normal levels (MDA: 17.2 nmol/g skin tissue; SOD: 46.8 U/g skin tissue; CAT: 53.2 U/g skin tissue) (*p* < 0.05), outperforming both GABA (MDA: 19.8 nmol/g skin tissue; SOD: 31.4 U/g skin tissue; CAT: 40.4 U/g skin tissue) and CSNPs (MDA: 22.8 nmol/g skin tissue; SOD: 32.8 U/g skin tissue; CAT: 46.6 U/g skin tissue) treated groups (Fig. 4A–C).

Following the same pattern, the PC rats developed elevated levels of pro-inflammatory cytokines, with IL-6 reaching 318.8 pg/g skin tissue, IL-1*β* reaching 117.4 pg/g skin tissue and TNF-*α* peaking at 753.2 pg/g skin tissue, approximately 2 folds higher than healthy controls (HC: 107.4, 55.8 and 354.4 pg/g skin tissue, respectively) (*p* < 0.05). Treatment with GABA-CSNPs showed remarkable efficacy, completely normalizing IL-6 level (107.4 pg/g skin tissue), IL-1*β* levels (55.0 pg/g skin tissue) and substantially reducing TNF-α (552.8 pg/g skin tissue) compared to the PC group (*p* < 0.05) (Fig. 4D, E and F). These findings collectively indicated that GABA-CSNPs possessed superior in vivo anti-inflammatory and antioxidant properties compared to their individual components.

## Discussion

GABA with its amino and carboxylic functional groups is a key inhibitory neurotransmitter in the human central nervous system (Milon et al. 2024) and a non-protein amino acid that is widely dispersed in biological systems (Sarasa et al. 2020). Beyond its role as a neurotransmitter, GABA demonstrates a broad spectrum of biological effects, such as antidiabetic (Khachatryan et al. 2024), anticancer (Oketch-Rabah et al. 2021), antihypertensive (Heli et al. 2022; Tian et al. 2014), anti-inflammatory, antioxidant (Madbouly et al. 2024), antimicrobial, and anti-allergic activities (Ngo and Vo 2019).

CSNPs represent an eco-friendly nanomaterial platform with dual capabilities in drug delivery. These biopolymer-based nanoparticles can both encapsulate diverse therapeutic compounds and be chemically functionalized with targeting ligands for cell-specific membrane recognition (Peers et al. 2022). This dual functionality enables safer, more precise cellular entry while significantly improving drug delivery efficiency and controlled release kinetics. The CSNPs enhance drug permeation through dual transport mechanisms: (1) transcellular uptake and (2) paracellular passage via reversible modulation of epithelial tight junctions. Their cationic character promotes strong electrostatic binding with anionic mucosal surfaces, with additional stabilization through hydrophobic interactions, ionic bonding, and hydrogen bridge formation (Khalaf et al. 2023).

GABA-CSNPs represent an innovative drug delivery platform that combines the neuro-immunoregulatory properties of GABA with the nanomaterial advantages of CS. Recent advances demonstrate their therapeutic potential in many applications including, treatment of liver-based diseases (J et al. 2012), hepatocyte regeneration (Shilpa and Nebu 2024), maintenance of glucose homeostasis and anti-diabetic therapies (Liu et al. 2018), renoprotection (Madbouly et al. 2024). The aim of this study is to report a novel antipsoriatic function of GABA-CSNPs in rats. In this work, to verify the activity of GABA-CSNPs, the effects of anti-inflammation and anti-oxidant were experimentally exploited.

In the present work GABA-CSNPs were usefully prepared via the ionic gelation method. In this method, the formation of GABA-CSNPs relies on electrostatic-driven self-assembly, initiated by CS protonation in acetic acid, which enables ionic crosslinking with the polyanion TPP. This interaction bridges adjacent CS chains via amino (NH₃⁺) groups, forming a stable nanoparticle matrix. GABA is encapsulated through physical entrapment within the CS-TPP network and electrostatic binding between its carboxylate group (COO⁻) and chitosan’s NH₃⁺ (Ramadan et al. 2022).

TEM analysis confirmed the successful formation of GABA-CSNPs with excellent physicochemical characteristics. The results revealed well-defined, spherical nanoparticles with a smooth surface morphology and a uniform size distribution, indicating a highly controlled and reproducible ionic gelation synthesis process. The formulated particles demonstrate remarkable uniformity with narrow polydispersity, which is favourable for biodistribution and cellular uptake efficiency. The discrete nature of the nanoparticles, with an absence of significant aggregation, benefits the stability of the colloidal suspension. This is attributed to the sufficient electrostatic repulsion between the positively charged CSNPs, which prevents coalescence and maintains their individual integrity (Marques Gonçalves et al. 2023). GABA-CSNPs exhibited a narrow size distribution, with diameters ranging from 49.7 to 55.3 nm. This small size is particularly advantageous for biomedical applications, as it facilitates enhanced cellular uptake and the potential to cross biological barriers like the skin or the blood–brain barrier (Antunes et al. 2021). DLS analysis confirmed the efficient loading of GABA into the CSNPs with average hydrodynamic diameter of around 57.63 nm. The final size of GABA-CSNPs remains within an optimal range for biomedical applications and potential delivery routes, including transdermal penetration or systemic administration, while avoiding rapid renal clearance. In a previous study, Liu et al. (Liu et al. 2018) also prepared GABA-CSNPs in a comparable particle size using the same method with average diameter about 50.4 ± 18.7 nm. Furthermore, GABA-CSNPs-based drug delivery system was prepared by Yoon and Kang (Yoon and Kang 2020) and Madbouly et al. (Madbouly et al. 2024) with particles of about 150 and 77.5 ± 16.5 nm, respectively.

Salt formation is the mechanism of interaction between GABA and CS via the bond formation between the NH₃⁺ group of CS and COO^−^ group of GABA. The DL% and EE% are critical parameters determining the therapeutic and translational potential of the NPs. The DL% shows the mass ratio of drugs to nanocarriers while the EE% quantifies the efficient use of substances throughout the manufacturing process. In general, the NPs preparation method and synthesis conditions greatly affect these values but it is more difficult to get high DL% than high EE% (Jiang et al. 2023; Herdiana et al. 2024). In the present work, the synthesized GABA-CSNPs demonstrated DL% of 26.60 ± 0.46 with EE% about 71.83 ± 1.26, indicating a highly efficient and promising system for GABA delivery. This result suggests that the synthesis protocol successfully incorporated a substantial mass of the active compound (GABA) relative to the carrier material (CSNPs). Shilpa et al. (Shilpa and Paulose 2014) reported EE% of 71 ± 7.2 using 20 μg GABA/ mL CS solution.

The in vitro release profile of GABA-CSNPs exhibited a characteristic biphasic pattern. This behaviour is consistent with established release mechanisms for hydrophilic drugs encapsulated in polymeric nanocarriers (Herdiana et al. 2023). The initial burst release phase (27.17% at 0.5 h) is primarily attributed to the immediate diffusion of GABA molecules adsorbed on or near the CSNPs surface. Such immediate diffusion is due to the high aqueous solubility of GABA and its relatively weak physical entrapment at the particle periphery. Following this, the release kinetics transitioned to a more gradual and sustained phase, reaching 77.83% at 4 h and eventually plateauing near 94.00% at 16 h. This sustained release phase is governed by a combination of factors: (1) the slower release of GABA from the denser core of the CSNPs, and (2) the progressive erosion and disintegration of the CSNP matrix itself, which is sensitive to the pH and ionic strength of the surrounding medium (Herdiana et al. 2022). The observed biphasic profile is highly advantageous for potential therapeutic applications. The initial burst release could facilitate a rapid onset of pharmacological action, which is desirable in treatments requiring immediate effects. The subsequent sustained release phase could then maintain therapeutic drug levels over an extended period (up to 16 h), potentially reducing dosing frequency. The drug release data of the GABA-CSNPs were fitted to numerous previous studies (Mujtaba et al. 2025; Alam et al. 2012). Shilpa et al. (Shilpa and Paulose 2014) observed a release pattern of 30 ± 2% in 0.5 h in GABA and 5-HT –CSNPs and the release continued up to 97 ± 8.9% at 20 h.

The remarkable stability of unloaded CSNPs, with a minor increase from 27.3 nm to 28.2 nm over three weeks, can be attributed to the strong, stable ionic cross-linking between CS and TPP. In a similar pattern, stability of GABA-CSNPs (from 57.6 nm to 58.3 nm) indicates that the encapsulated GABA does not promote particle aggregation or polymer matrix degradation over time. This suggests that GABA is not only superficially adsorbed but is incorporated within the matrix in a way that does not affect the CSNP’s core structure or its electrostatic stabilization. This high degree of physical stability benefits nanotherapeutic to possess a clinically relevant shelf-life without requiring complex storage conditions and to maintain their structural integrity long enough in the bloodstream or at a localized site of administration, ensuring optimal pharmacokinetics and biodistribution (Nagati et al. 2022).

The zeta potential measurements provided important understandings into the surface properties and colloidal stability of the formulated GABA-CSNPs. The significant enhancement in positive surface charge from + 18.76 mV for unloaded CSNPs to + 35.93 mV for GABA-CSNPs is a direct consequence of successful GABA encapsulation. The effective interaction between CS’s amine groups and GABA’s carboxyl group amplified the overall positive charge on the formulation. According to the present data, zeta potential of GABA-CSNPs was stable over the 21-day evaluation period ensuring the maintenance of strongly positive values and strong electrostatic repulsion between particles GABA-CSNPs suspension that are stable and resistant to aggregation.

The antioxidant capacity of GABA-CSNPs to scavenge DPPH free radicals is estimated as colour change of DPPH from purple into a yellow solution at 517 nm absorbance. All the tested formulations showed antioxidant ability with different degrees. However, free GABA and unloaded CSNPs were less antioxidants than GABA-CSNPs. As GABA-CSNPs formulation maintained remarkable antioxidant activity even at lower concentrations (31.25–125 μg/mL), we can conclude its significantly higher hydrogen-donating ability. The antioxidant activity of CS and its formulation was linked, in many studies, to the capacity of active hydroxyl and amino groups that can absorb a hydrogen ion to form ammonium (NH3 +) groups (Chen et al. 2019a). As high degree of deacetylation increases the number of amino groups, thereby amplifying the hydrogen‐donating ability of the antioxidants (Ashoush et al. 2022). Also, previous hypothesis related the lowered molecular weight polysaccharides to the higher concentration of reductive hydroxyl groups per mass unit. Consequently, the enhanced DPPH scavenging power of CS-based nanoformulations (Zhang et al. 2013). Based on the present results, encapsulation process not only preserved but potentially enhanced the biological efficacy of GABA and its applications where oxidative stress is a factor. These findings highlighted the potential of GABA-CSNPs as an effective antioxidant delivery system, with the CS carrier contributing to both stabilization and improved performance of the incorporated GABA.

Notably, GABA-CSNPs demonstrated enhanced cytotoxicity profiles compared to both free GABA and CSNPs at equivalent concentrations, suggesting that the encapsulation process did not introduce toxic effects but even increased its safety and lowered its IC50 concentration. As, the IC50 of GABA-CSNPs is more than 1.9 times higher than that of GABA and over 2.9 times higher than that of CSNPs. This means enhanced biocompatibility for potential therapeutic use based on this GABA-CSNPs formulation. These results confirmed the excellent biocompatibility of the GABA-CSNP formulation, supporting its potential for therapeutic applications where minimal cellular toxicity is essential. The presence of positive charge and high cell binding affinity of CS may enhance the exposure of GABA to the internal body environment and hence enhanced biocompatibility (J et al. 2012). In consistency with the present findings, Tolstova et al. (Tolstova et al. 2023) confirmed the efficacy of CS and CS-based formulations in tissue repairing. Additionally, a systematic literature review by Frigaard et al. (Frigaard et al. 2022) examined 70 articles to assess the cytotoxicity of CSNPs, concluding that low cytotoxicity was observed regardless of the specific particle composition or the assay and cell line used. This finding, while generally comforting, was associated with a recommendation that all new CS-based formulations undergo rigorous characterization and cytotoxicity testing before commercialization. Shilpa et al. (J et al. 2012) reported that GABA-CSNPs increased cell regeneration of cultured hepatocytes. In a prior study, we found that decorating CSNPs with GABA improved cell survival rates due to the compound’s antioxidant effects (Madbouly et al. 2024).

The membrane stabilization assay provided evidence for the enhanced anti-inflammatory properties of GABA when loaded on CSNPs. Notably, the nanoparticle formulation maintained significant anti-inflammatory effects even at lower concentrations (100–400 μg/mL), suggesting that CS encapsulation not only preserved but possibly potentiated GABA’s therapeutic properties. The activity of GABA-CSNPs is competitive with a powerful standard anti-inflammatory drug like Indomethacin, strongly supporting its potential as a natural and effective alternative for managing inflammation-based conditions. Early studies also confirmed the in vitro anti-inflammatory enhanced properties of CSNPs-based formulations (Tawfik and Farid 2025; Tarek et al. 2025; Ashraf et al. 2025).

From the previously discussed in vitro studies, we confirmed the benefits of loading GABA on CSNPs on its bioavailability, antioxidant and anti-inflammatory abilities. The enhanced efficacy of GABA-CSNPs stems from a synergistic two-part mechanism where the CSNPs vehicle first dramatically improves GABA’s delivery and bioavailability through mucoadhesion, tight junction opening, surface area of contact with the absorption site and enhanced endocytic cellular uptake, leading to high intracellular concentrations (Herdiana et al. 2023; Saleh et al. 2022). Once effectively delivered on CSNPs, the GABA then achieves a powerful pharmacological functions by directly scavenging free radicals to reduce oxidative stress, stabilizing cell membranes and inhibiting pro-inflammatory cytokines to suppress inflammation, and modulating immune cell function via GABA receptors to promote an anti-inflammatory state (Zhu et al. 2019; Deng et al. 2013).

Over the course of ten days, IMQ was used topically twice a day to induce PS-like condition. The dorsal skin of administrated rats developed scaly, inflammatory lesions resembling plaque-type psoriasis when IMQ was applied daily (Khalil et al. 2025). Severe scaling and erythema in the PC group demonstrated the successful establishment of the IMQ-induced PS rat model. These psoriatic symptoms were considerably reduced by GABA or CSNPs single treatment. The GABA-CSNPs combination therapy, on the other hand, showed the best, synergistic impact, reducing inflammation and improved the psoriasis pathology score, reduce local scale production and skin thickening of Aldara®-induced psoriasis-like skin lesion to a degree that was quite similar to the HC group. This strong antipsoriatic impact is probably due to the CSNPs system’s improved, targeted administration of immunomodulatory GABA effects.

Oxidative stress is a deleterious factor affecting PS pathogenesis. Patients with psoriasis develops a redox imbalance in serum, plasma and skin as reduced antioxidants (SOD, CAT and vitamin E) and elevated MDA and hydroperoxides (Hu et al. 2022). This intensified oxidative state drives epidermal hyper-oxidation and abnormal proliferation of keratinocytes. In response, these dysfunctional cells secrete a cascade of inflammatory mediators, autoantigens, and cytokines, which then recruit and stimulate the maturation of dendritic cells, thus amplifying the inflammatory cascade in psoriatic lesions (Wu et al. 2025).

In the present study MDA, SOD and CAT were assessed in skin tissue as key biomarkers of oxidative stress. The success of our PS-like model was confirmed in the PC group that showed a state of high oxidative stress, with elevated damage (MDA) and depleted antioxidant defences (SOD, CAT). The decline in antioxidant systems was linked to the overexpression of inflammatory mediators, which increase in lipid peroxidation processes, thus continuing this cycle (Wu et al. 2025). Numerous earlier studies highlighted the correlation between MDA, SOD and CAT and the severity of PS expression (Dobrică et al. 2022; Pujari et al. 2014; Gabr and Al-Ghadir 2012). By simultaneously reducing oxidative damage (lowered MDA) and boosting the skin’s intrinsic antioxidant antioxidant defences (elevated SOD and CAT), GABA-CSNPs treatment maximally alleviated the PS-mediated oxidative damage. This hypothesis is supported by earlier studies that described the GABA’s protective role by reducing cellular ROS and MDA levels (Tang et al. 2018; Hou 2011; Kurjak et al. 2011).

As a chronic, immune-mediated inflammatory and hyperproliferatory skin disorder, PS, involves dysregulated interactions between the innate and adaptive immune systems, notably involving a characteristic shift towards Th1-dominant cytokines (Pomi et al. 2025). These cytokines are central players in the "psoriatic cascade," driving keratinocyte proliferation, skin inflammation, and the recruitment of other immune cells (Han et al. 2025). Our finding revealed dramatic elevations of Th1 cytokines as IL-6, IL-1*β* and TNF-*α* in skin tissue from PC group as a classic hallmark of the psoriatic inflammatory microenvironment.

Unloaded CSNPs showed a modest regulatory effect on cytokine production, due to chitosan’s known bioactivity. In addition, free GABA shows a stronger anti-inflammatory effect. However, the GABA-CSNPs combination is profoundly more effective in counteracting of Aldara®, indication a synergistic suppression of Th1 inflammation (Khalil et al. 2025 The nanoparticle system ensures a high concentration of GABA is delivered to the inflamed skin tissue, where it can maximally inhibit Th1 cytokine production as a reflected efficacy to down regulate IL-6, IL-1*β* and TNF-*α*.

The GABA receptors (R) (GABA_A_, GABA_B_ and GABA_C_) are key regulators of inflammation in psoriatic lesions, due to their expression on immune cells (D’Hulst et al. 2009). Upon activation on the macrophages and CD4 T cells, GABA/GABA _A_ axis inhibits T cell proliferation and reduces the secretion of inflammatory mediators like IL-6, IL-12, iNOS, IL-1*β*, and TNF-*α* (Wu et al. 2017). Accordingly, Th1 cytokines were primarily inhibited by activating GABA A-R activation mitigates cutaneous inflammation, promotes healing, and prevent epidermal hyperplasia in models of skin injury, highlighting its potential relevance to PS (Tian and Kaufman 2023; Nigam et al. 2010). In addition, the functioning of GABA/GABA _B_ axis of neutrophils allows GABA to act as a chemoattractant and promotes directed migration to sites of inflammation and injury (Kniazeff 2020). Nigam et al. (Nigam et al. 2010) revealed a marked increase in GABA and GABA_A_R expression within psoriatic lesions. The GABA and GABA_A_ receptor-positive cells were mainly: A] macrophages located near the papillary dermis, B] CD4 helper T Cells that produces IFN-γ, IL-17 and other inflammatory mediators and c] epidermal neutrophils. Chen et al. (Chen et al. 2019b) also confirmed inhibition of the T cell proliferation which is linked to inflammatory diseases after activation of GABA receptors. GABA proven efficacy in prevention of stress immune signalling via elevation of IL-2, IL-10, and IFN- γ as well as lower levels of nuclear factor-kappa B (NF-КB), IL-1*β*, and TNF-*α* (Chen et al. 2021).

## Conclusion

This study successfully developed, characterized, and validated GABA-CSNPs as a novel nanotherapeutic formulation demonstrating significant potential as an antipsoriatic agent. Using the ionic gelation method GABA-CSNPs developed optimal physicochemical properties for drug delivery, including a uniform, spherical morphology, a small hydrodynamic diameter, a high positive zeta potential and excellent stability. GABA-CSNPs exhibited high EE% and favourable biphasic drug release profile. In vitro studies confirmed improved biocompatibility with superior antioxidant and anti-inflammatory capacity of GABA-CSNPs. The in vivo efficacy was demonstrated in an IMQ-induced PS rat model. GABA-CSNPs provided pronounced therapeutic recovery, expressed as normalized psoriatic skin morphology, restored epidermal architecture, and reversed key pathological markers. This antipsoriatic capacity of GABA-CSNPs was achieved through a dual mechanism of oxidative amelioration and suppression of Th1 pro-inflammatory cytokines.

## Data Availability

No datasets were generated or analysed during the current study.
